# A prospective pilot study to evaluate an animated home-based physical exercise program as a treatment option for patients with rheumatoid arthritis

**DOI:** 10.1186/s12891-016-1208-3

**Published:** 2016-08-18

**Authors:** Jan Zernicke, Claudia Kedor, Angela Müller, Gerd-Rüdiger Burmester, Anett Reißhauer, Eugen Feist

**Affiliations:** 1 Department of Rheumatology and Clinical Immunology, Charité University Medicine, Chariteplatz 1, 10117 Berlin, Germany; 2 Charité Health Care Center for Physical Medicine, Chariteplatz 1, 10117 Berlin, Germany

**Keywords:** Rheumatoid arthritis, Exercises, Physical therapy, Rehabilitation, Patient perspective, Wii® game console

## Abstract

**Background:**

Physical exercises and physiotherapy are of great importance for maintenance of joint function in patients with rheumatoid arthritis (RA). However, many RA patients complain about problems to receive prescriptions or have a lack of access to physiotherapy. Recent reports have shown positive effects of the Wii game console on physical and psychosocial conditions of patients with other underlying diseases. The primary objectives of this prospective controlled pilot study were to investigate feasibility and patients’ assessment using an animated home-based exercise program.

**Method:**

This pilot study was conducted as a single-center, cross-over trial with two treatment arms over 24 weeks. Eligibility criteria included patients with RA reaching low disease activity under therapy with a biological disease modifying anti-rheumatic drug (bDMARD). After detailed instruction, 15 patients started with a conventional home-based physical exercise program and 15 patients began with a predefined animated exercise program by using the Wii game console for 12 weeks. Afterwards, patients were crossed-over to the other treatment arm for another period of 12 weeks.

Multi-methodical assessments were performed by qualitative analysis of the interview-data as well as statistical analysis of functional tests and patient reported outcomes (PRO’s).

**Results:**

Evaluation of the interviews indicated feasibility and usefulness of the chosen animated home-based exercise program. Forefoot disabilities were identified as a main limiting factor for performing some of the animated exercises. After 12 weeks, both treatment arms showed improvement of functional tests without significant differences between groups: Overall muscle strength improved for a mean value of 10 Newton (+12 %) and the mean 6-min walk test (6-MWT) distance increased for 28 meters (+5 %).

**Conclusion:**

This study showed that an animated home-based exercise program by using a Wii game console was feasible and beneficial for RA patients. Compared to standard physical home exercises, similar effects were observed indicating that such an animated program might be an alternative supportive option for RA patients.

**Trial registration:**

ClinicalTrials.gov ID: NCT02658370 (19-Jan-2016).

**Electronic supplementary material:**

The online version of this article (doi:10.1186/s12891-016-1208-3) contains supplementary material, which is available to authorized users.

## Background

Rheumatoid arthritis (RA) is an autoimmune disease with a prevalence of approximately 0.5 % in central Europe [1, 2]. The economic impact of RA is significant for the society as well as for the individual taking into account both direct and indirect costs [3]. The effectiveness of conventional and biologic disease modifying anti-rheumatic drug (DMARD) therapy has improved considerably the treatment outcomes in RA over the past 15 years [4, 5]. However, despite excellent medical treatment options, a high proportion of RA patients still complains about loss of physical function [6–8].

Hence, physical exercises and physiotherapy are of high importance for maintenance of joint function in patients with rheumatoid arthritis. International guidelines for long-term treatment of RA also recommend non-pharmacological interventions as adjunctive therapy to pharmacologic treatment [9, 10]. In this context, many publications underline that physical therapy plays an important role regarding preventative, curative and rehabilitative aims in the treatment of RA patients. Especially patients with progressive and aggressive arthritis benefit from physiotherapy and physical exercises. These RA patients can delay or even avoid an early invalidity as well as improve their fitness [11–16].

Following these recommendations and guidelines, RA patients should be encouraged to be as physically active as possible. Even high-intensity exercises have been described as safe and effective for RA patients without increasing disease activity. In this setting, rheumatologists and physical therapists only have to take into account individual patient requirements and current joint damage before approving weight bearing and/or high-intensity exercises [17–22].

Furthermore, arthritis patients should try different activities to find those they enjoy and to have a variety of physical activity options [23]. Development and evaluation of programs and strategies to promote long-term, self-directed physical activity in community-based settings are recommended for future research [24]. However, many RA patients report about a lack of access to physical therapy and supervised community-based exercises.

By considering these difficulties, an animated physical exercise program using a game console could be an alternative approach. The exercise-based game software Wii-fit for the Nintendo™ Wii game console was released in 2007 and has generated a specific interest of investigation. Recent publications have shown positive effects of the Wii activity games and exercises on physical and psychosocial conditions of patients with different underlying diseases. The results of a randomized cross-over trial of children with movement difficulties appreciate the use of Wii fit within the therapeutic programs. [25] To quote another example, present results support the use of the Wii-fit program as a feasible, safe and potentially effective therapeutic tool to augment the rehabilitation of young children with developmental delay [26]. The exercise based game-software also demonstrated positive outcomes at the impairment and functional levels of a person with cerebral palsy [27]. It had been illustrated in a case study that the use of Nintendo Wii™ fit training and body weight support were effective interventions to achieve functional goals also in elderly patients with lower limb amputation [28].

Taken together, this tool has inspired the use especially in the domain of neuro-rehabilitation as a training device for balance ability. From 2007 to 2010 only ten Wii-fit balance related scientific papers have been published on the medical usefulness of the Wii-fit program, but afterwards the number of papers has shown an exponential increase [29]. In the field of osteology, this animated program has been investigated only once showing beneficial results of balance training in patients with osteoporosis [30]. Consequently, it shows that the Wii console can be a serious object of investigation and a valid instrument [31, 32]. In fact, a home-based animated physical exercise program could be also cost-effective. It is freely available and adjustable to the individual situation.

In this pilot study, we aimed to investigate at first the feasibility and patients acceptability of a mainly home based animated physical exercises program in RA in order to potentially proof the concept. Furthermore, different physical outcome measures and PRO’s were captured to allow a comparison of treatment effects and estimation of needed sample size for further studies.

## Methods

This pilot study was conducted as an investigator initiated, not manufacturer supported, single-center, cross-over trial with two treatment arms over 24 weeks. Six game consoles were purchased and distributed for free to the patients.

Patients fulfilled the 1987 and 2010 American College of Rheumatology (ACR) criteria for RA [33, 34]. Inclusion criteria included patients achieving a self-reported disease activity (patients’ global assessment, PtGA) < 30 mm under therapy with a biological DMARD therapy according to label. Thus, all patients were inadequate responders and/or showed intolerance to conventional DMARDs [cDMARDs]).

Patients only on cDMARD therapy were excluded with the aim to investigate a homogeneous cohort with established and advanced RA. We addressed the question whether such a cohort could further benefit from standard physiotherapy in comparison to an animated exercise program. Key exclusion criteria were epilepsy, flare of RA and a previous use of a Wii console for more than 5 h. To ensure standardized instruction for the animated exercise program, this was a mono-centric study and all participants were recruited from the out-patient department of the Department of Rheumatology at Charité University Hospital Berlin. The study has been approved by the Ethics Committee of the Charité university hospital in Berlin. All subjects’ written consent was obtained according to the Declaration of Helsinki.

### The console – tool of investigation

The Wii console by Nintendo™ is a home video game console. The primary controller for the console (Wii Remote) can be used as a handheld pointing device which detects movement in three dimensions. It uses a combination of built-in accelerometers and infrared detection to sense its position in 3D space, when pointed at the LEDs in the sensor bar, which is located on the top of the TV. This allows users to control the game with physical gestures as well as button activation [35, 36].

Wii fit plus, the software we used for this study, was released in 2009 and contains more than 50 exercises and activity games. The exercises are separated into four categories: Yoga, muscle strength, balance games and aerobic [37, 38].

The fitness software Wii-fit plus comes together with a balance board. This peripheral device is like a scale with four sensors (left side/right side and front/back) and looks most similar to a step-board. The Balance board was found to be a valid tool for assessing standing balance [32] and is also sensitive for lateral movements and shifting of weight on the toes or heel. In general, the Wii console is an easy, rather inexpensive and intuitively to use device which does not require a special education. Furthermore, the integrated tutorial is sufficient to guide the user through the menu.

#### Treatment plan

Fifteen patients started with the animated exercise program by using the Wii game console (Wii-group) for 12 weeks and 15 patients started with a conventional home-based physical exercise program (PT-group). Afterwards, patients were crossed-over to the other treatment arm for another period of 12 weeks. The recruitment strategy comprised informational events for the local patient groups as well as presentations at public meetings. The highest number of attendees was recruited from the outpatient unit of the rheumatology department at the Charité. Patients were enrolled in an alternate mode and at the beginning of each program, the participants were instructed in detail. The patients were asked to participate in 2 or 3 supervised sessions (approximately 1 h/session) in order to rehearse the exercises of the conventional physical exercise program or to become acquainted with the game console, respectively. At the end of the introduction phase, all patients received a manual, which was conducted in cooperation with physiotherapists. According to the training schedule, every patient was stimulated to exercise 3 times a week for approximately 30 min/session.

### Approach in the Wii-group

Before study start, the exercises and activity games of the Wii™ fit plus software were judged by physiotherapists and the study group in order to exclude such exercises in advance, which do not qualify for RA patients. As a result, the manual for the Wii-fit exercise program comprises five illustrations (screenshots) of the software menu, where improper exercises were crossed out. In more specific terms, this means that patients could select from at least 12 Yoga exercises, 11 muscle-strength exercises, 7 balance games and 6 aerobic exercises. For the duration of the study, patients in the Wii group loaned a commercially available game console (Wii-console) together with the Wii-fit software and the balance board for use at home. The intention of this study group was that patients were allowed to choose the exercises by themselves, but with the obligation of doing at least two exercises from each category.

### Approach in the PT-group

In preparation of this study a compilation with 31 exercises especially for RA was developed by physiotherapists and the study group. The conventional home-based physical exercise program was also divided into four categories: strength training (10 exercises), coordination (2), joint mobility (10) and relaxation (9). The ambition of this treatment arm was to reflect the routine of a home-based physical exercise program as exact as possible. First and foremost, the physiotherapists assessed the patients status during the first session of the introduction phase (i.e. stage of RA, joint mobility, age, deficiency of muscle strength). Secondly, the physiotherapists chose individual exercises from the study specific compilation and practiced each exercise with the patient. Finally, every patient in the PT-group received a manual with 10 to 12 exercises, adapted to his/her individual needs. Intensity, rest interval and repetitions of the exercises were not pre-defined with respect to the diversity of the participants and to avoid therapeutic problems.

A multi-methodical approach was used for this study to determine effects and estimate feasibility: analysis of qualitative and quantitative data.

#### Qualitative data collection

Qualitative research approach in its diversity is a meanwhile accepted approach in order to understand the *why* and *how* of human actions. Both qualitative and quantitative methods complement each other, including medicine [39]. For this trial, we chose the approach of a qualitative data analysis (QDA), in detail a ‘*summarizing qualitative content analysis’* of interviews [40, 41].

Semi-structured, face to face interviews were performed and recorded at baseline, week 12 and week 24. The decision to use semi-structured interview technique was based on the opportunity for both interviewer and interviewee to discuss some topics in more detail. With open ended questions the patients were asked to explain their experiences of the last 12 weeks. In addition, a series of predefined questions helped the interviewee to express his/her experience. All interviews were transcribed into a written form and patient’s explanations were analyzed by using special software for coding patient’s response (open code 4.0.1 [42]).

The analysis of interview material followed three steps: First, transcripts were organized, reduced and prioritized to identify issues and ideas that are relevant to the focus of the evaluation. Second, the words and phrases of each patient, which were used to describe the experience with the exercise program, were extracted and coded. As a third step, an inter-individual comparison was done by structuring and grouping of similar kinds of codes into categories. These categories were developed exclusively on the basis of the interview data in an inductive approach [40]. The codes as well as categories were discussed and revised continuously by the study group (2 physicians, 1 sports scientist) in order to increase the inter-individual consensual comprehension and to avoid counterproductive coding.

Due to its nature, these qualitative data do not qualify for statistical analysis but are useful for evaluation of feasibility.

### Definition of high-responder, low-responder & non-responder

Patients’ response was judged on the basis of the interviews and the consoles diary function. Participants were rated as high-responders if a high motivation was expressed at the end of each treatment arm according to the interview. In addition, high-responders were evaluated as overachievers by diary and/or described an interest in continuation of the exercise. A patient was classified as a low-responder, if the training schedule was met and a neutral point of view displayed regarding the exercise program. Non-responders were defined as underachiever by practicing less than two times a week and expressing a low level of motivation.

### Patient reported outcomes (PRO’s)

Patient reported outcomes cover 3 domains and were assessed at baseline, week 12 and week 24 visits: physical function was measured using the Health Assessment Questionnaire (HAQ-DI), disease activity was evaluated using patient’s global assessment on a 100 mm visual analogue scale (PtGA) and quality of life was assessed using the Short Form 36-Item (SF36) questionnaire [43, 44].

#### Physical function tests

At Baseline, week 12 and week 24 visits, nine muscle groups were assessed using a hand held dynamometer (isometric measurement). Measured muscle groups included neck extensors, neck flexors, shoulder abductors, elbow extensors, elbow flexors, 3-point grip strength, hip flexors, knee extensors and knee flexors. The mean value of a triple measurement of each muscle group was used for further calculation. The hand held dynamometer (CITEC by CIT Technics) had been utilized before in clinical trials to investigate myositis and has been described as a reliable tool [45].

Changes of physical status were further assessed using respiratory function test. At each visit, the peak expiratory flow was taken by a peak flow meter (Vitalograph® peak flow meter by Vitalograph, Ireland). The mean value of a triple measurement was used for further calculation. A change of +8 % and -11.8 % was defined as being medically important [46]. At the end of each visit, the patients were asked to perform a six-minute walk test (6-MWT). The 6-MWT is an appropriate tool to assess the submaximal level of functional performance and is utilized in many conditions [47]. A MCID of 20 meters have been specified for this study [48, 49].

#### Statistics

To compare both groups statistical analyses were performed with SPSS (Version 20.0.0.1) using the ‘Analysis of Covariance’ (ANCOVA), adjusted for baseline status. Due to the fact of different pre-conditions at the beginning of the second treatment period (after cross-over), we focused on statistical ‘head to head’ analysis of the week 12 results. A paired *t*-test was used to compare mean changes of the cohort.

## Results

A total of 30 RA patients (25 females, 5 males) with a mean age of 56 (SD ± 9) years were enrolled. The mean disease duration was 13 (SD ± 9) years at baseline. 12 patients were currently treated with Rituximab, 10 patients with Tocilizumab, 5 with Abatacept, 2 with Etanercept and 1 patient was treated with Certolizumab during the study. 21 patients (70 %) had been treated before with another bDMARD, 15 patients (50 %) with ≥2 other bDMARDs. Mean patients VAS was 16.8 mm (SD ± 8) at baseline with a corresponding DAS28 of 2.8 (SD ± 1.2). The mean DAS28 slightly decreased at week 12 visit (-0.2) but returned to baseline level at the end of the study. The detailed baseline characteristics are shown in Table 1. Three female patients dropped out prior to the cross-over visit. Two of these patients dropped out due to motivational reasons during the study phase with conventional home-based physical exercises. One patient complained about lack of time to use the Wii console.

**Table 1:** Baseline characteristics of patients

	Total	Patients starting with animated exercise program (Wii console)^a^1	Patients starting with conventional home-based physical exercises^a^1
Number of patients, n	30	15	15
Age (years), mean (SD)	56 (±9)	52 (±8)	59 (±9)
Age (years), min.	34	34	45
Age (years), max.	69	65	69
Age > 60 years, n (%)	11 (37 %)	3 (20 %)	8 (53 %)
Age 50 – 60 years, n (%)	10 (33 %)	7 (47 %)	3 (20 %)
Age <50 years, n (%)	9 (30 %)	5 (33 %)	4 (27 %)
Female, n (%)	25 (83 %)	10 (67 %)	15 (100 %)
Disease duration (years), mean (SD)	13 (±9)	10 (±7)	16 (±9)
disease duration (years), max.	36	26	36
disease duration (years), min.	1	1	1
DAS28, mean (SD)	2.8 (±1.2)	3.0 (±1.5)	2.6 (±0.9)
DAS28 score, max.	5.3	5.3	4.4
DAS28 score, min.	0.5	0.9	0.5
HAQ-DI score, mean (SD)	0.85 (±0.53)	0.72 (±0.52)	0.98 (±0.51)
HAQ-DI score, max.	1.75	1.75	1.63
HAQ-DI score, min.	0.00	0.00	0.00
Patient’s VAS disease activity, mm	17	16	18
Current biological DMARD therapy			
Rituximab, n (%)	12 (40 %)	7 (47 %)	5 (33 %)
Tocilizumab, n (%)	10 (33 %)	5 (33 %)	5 (33 %)
Abatacept, n (%)	5 (17 %)	2 (13 %)	3 (20 %)
Etanercept, n (%)	2 (6 %)	1 (7 %)	1 (7 %)
Certolizumab, n (%)	1 (3 %)	0 (0 %)	1 (7 %)
Previous biological DMARD therapy			
Rituximab, n (%)	4 (13 %)	2 (13 %)	2 (13 %)
Tocilizumab, n (%)	3 (10 %)	1 (7 %)	2 (13 %)
Abatacept, n (%)	2 (6 %)	1 (7 %)	1 (7 %)
Etanercept, n (%)	10 (33 %)	7 (47 %)	3 (20 %)
Certolizumab, n (%)	1 (3 %)	1 (7 %)	0 (0 %)
Golimumab, n (%)	1 (3 %)	0 (0 %)	1 (7 %)
Secukinumab, n (%)	1 (3 %)	1 (7 %)	0 (0 %)
Infliximab, n (%)	6 (20 %)	2 (13 %)	4 (27 %)
Anakinra, n (%)	1 (3 %)	1 (7 %)	0 (0 %)
≥ 3 previous biological DMARD’s, n	7 (23 %)	4 (27 %)	3 (20 %)
≥ 2 previous biological DMARD’s, n	8 (27 %)	4 (27 %)	4 (27 %)
only 1 previous biological DMARD, n	6 (20 %)	2 (13 %)	4 (27 %)

^a^1 with a cross-over to the other treatment arm after 12 weeks SD, standard deviation; min, minimum; max, maximum; HAQ-DI, health assessment questionnaire disability index; DAS28, disease activity score using the 28 joint count; DMARD, disease modifying anti-rheumatic drug; VAS, Visual analogue scale

### Results of the interview analysis

**Fig. 1:**
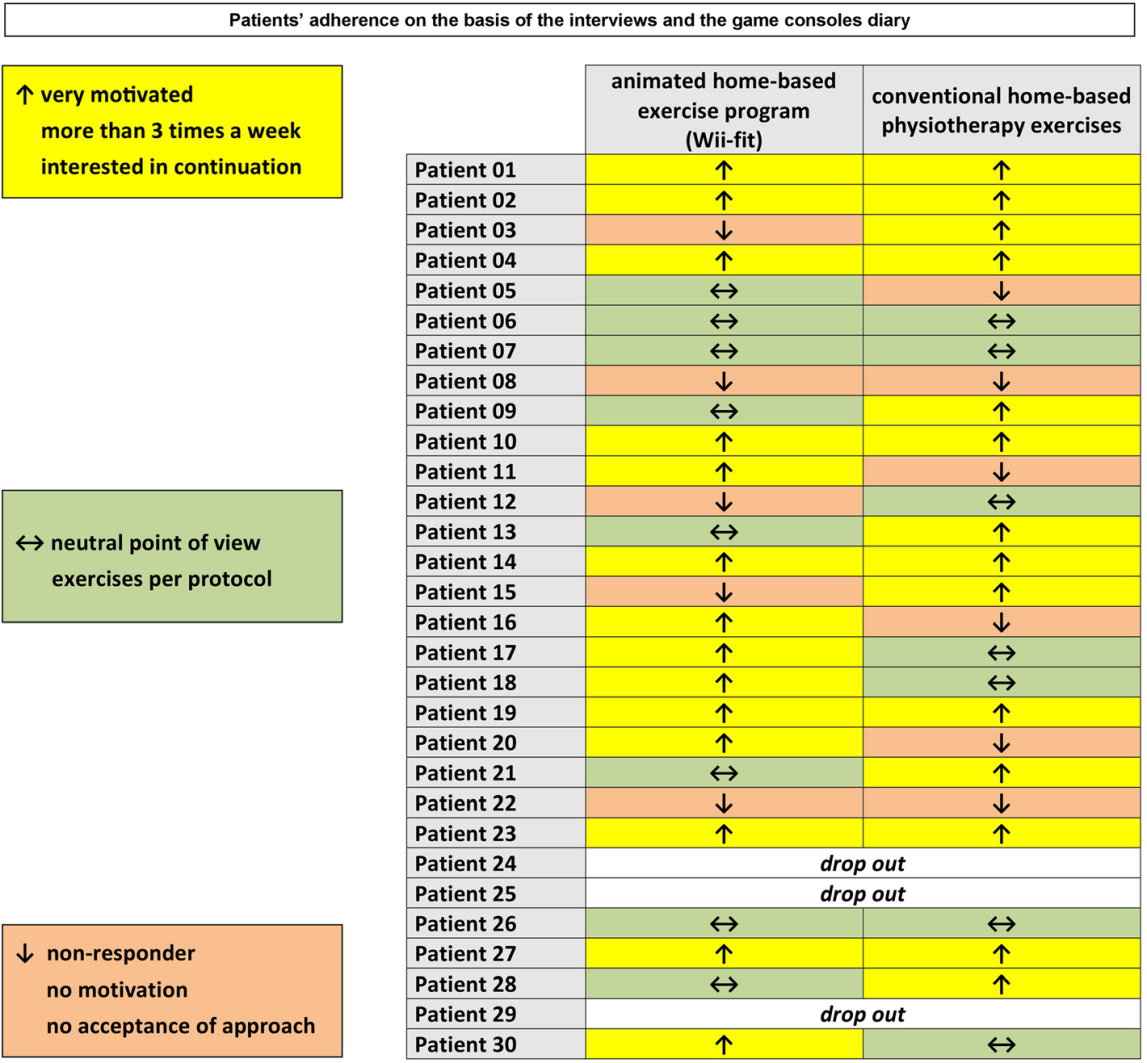
Patients’ response to the exercise program

More than 22 h of interview data were transcribed and analyzed with a mean duration of 15 min of each interview (SD ± 7). The assessment of patient’s response showed that about half of the participants in each group were motivated and adherent to the exercise with very little difference between groups. The highest proportion (14 patients in each group), had trained more than three times a week and was very interested in continuing (Fig. 1).

**Fig. 2:**
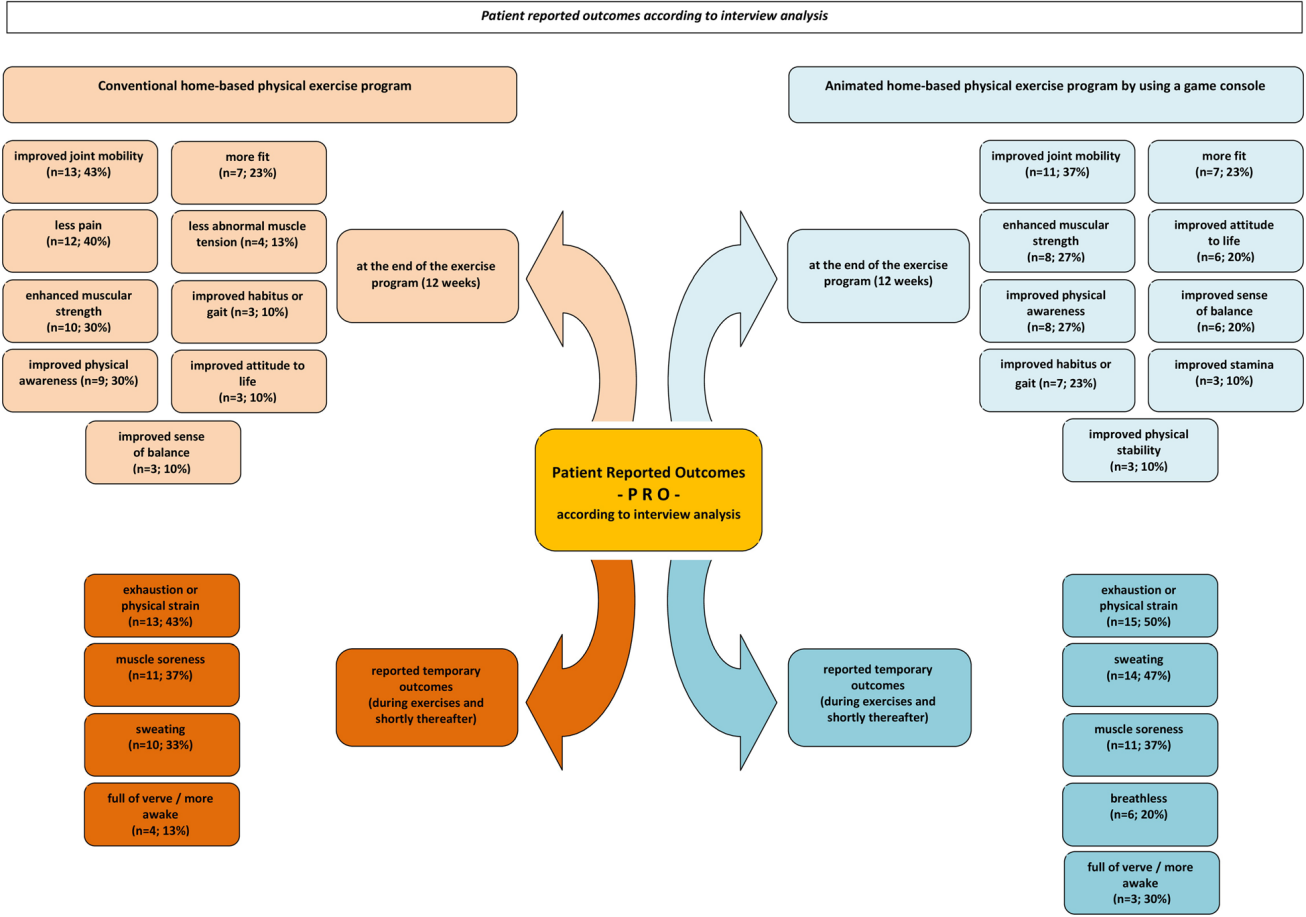
Patients reported outcomes according to interview analysis

**Fig. 3:**
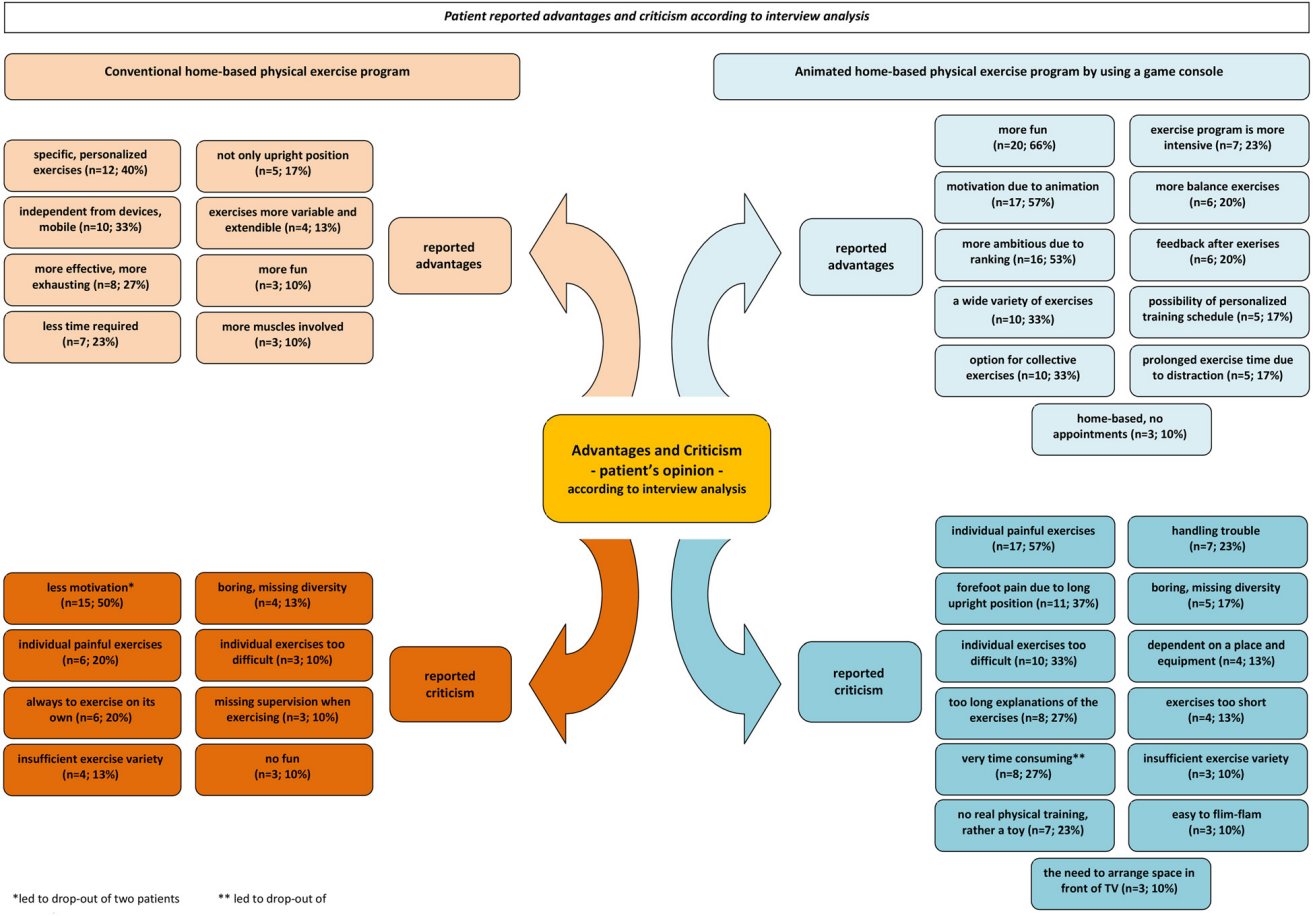
Patients reported advantages and criticism according to interview analysis

The results of the interview analysis were divided into 2 categories: patients reported effects (Fig. 2) and points of advantages as well as criticism of the respective treatment forms (Fig. 3). These schemes are to be understood as the main result of the extensive qualitative content analysis of the interviews.

#### Reported treatment effects

With respect to most frequent accompanied events (in a positive manner), patients in both treatment arms described exhaustion or physical strain (PT-group 43 %; Wii-group 50 %), sweating (PT-group 33 %; Wii-group 47 %), muscle soreness (PT-group 37 %; Wii-group 37 %) and the feeling to be more awake (PT-group 13 %; Wii-group 10 %) as effects during the exercises and shortly thereafter (short-time effects). On the other hand, patients also reported improved joint mobility (PT-group 43 %; Wii-group 37 %), enhanced muscular strength (PT-group 33 %; Wii-group 27 %), improved physical awareness (PT-group 30 %; Wii-group 27 %) and improved sense of balance (PT-group 10 %; Wii-group 20 %) as effects at the end of each treatment (long-time effects). The reported feedback from both groups is comparable without relevant differences. The ability to induce all these effects was reported by patients in both groups (Fig. 2).

#### Advantages and criticism explained by the Wii-group

According to patients feedback in interviews the most frequently explained advantages of the animated exercise program were the following: to have more fun (66 %), to raise the motivation due to animation (57 %), to have more ambition due to ranking of the results (53 %) and to have a wide variety of exercises (33 %). Individual painful exercises (57 %), individual exercises too difficult (33 %) and time consuming explanations of the exercises (27 %) were the essential reported points of criticism of the animated exercise program. Mainly forefoot disabilities were identified as limiting factor for performing some of the exercises, when standing on the Wii Balance-Board (37 %). These patients with forefoot deformities reduced the practice time or ignored these individual exercises.

Despite of the criticism and disadvantages, all patients expressed a general feasibility and easy handling of the game console for RA patients. The motivating aspect of the game console plays an important role independently of patients’ age and disease duration. Many patients stated a positive influence by the game console on their perseverance during the exercising time. More details are shown in Fig. 3.

#### Advantages and criticism explained by the PT-group

In contrast, items like specific and personalized exercises (40 %), independency from devices (33 %) as well as being more effective (27 %) were the most commonly reported advantages of the conventional physical exercise program. Less motivation (50 %), the fact of performing the exercises alone (20 %) and an insufficient variety of the exercises (13 %) can be interpreted as key disadvantages of the conventional home-based exercise program according to the interview analysis. Individual painful exercises were also described in this group (20 %).

### Test results

**Fig. 4:**
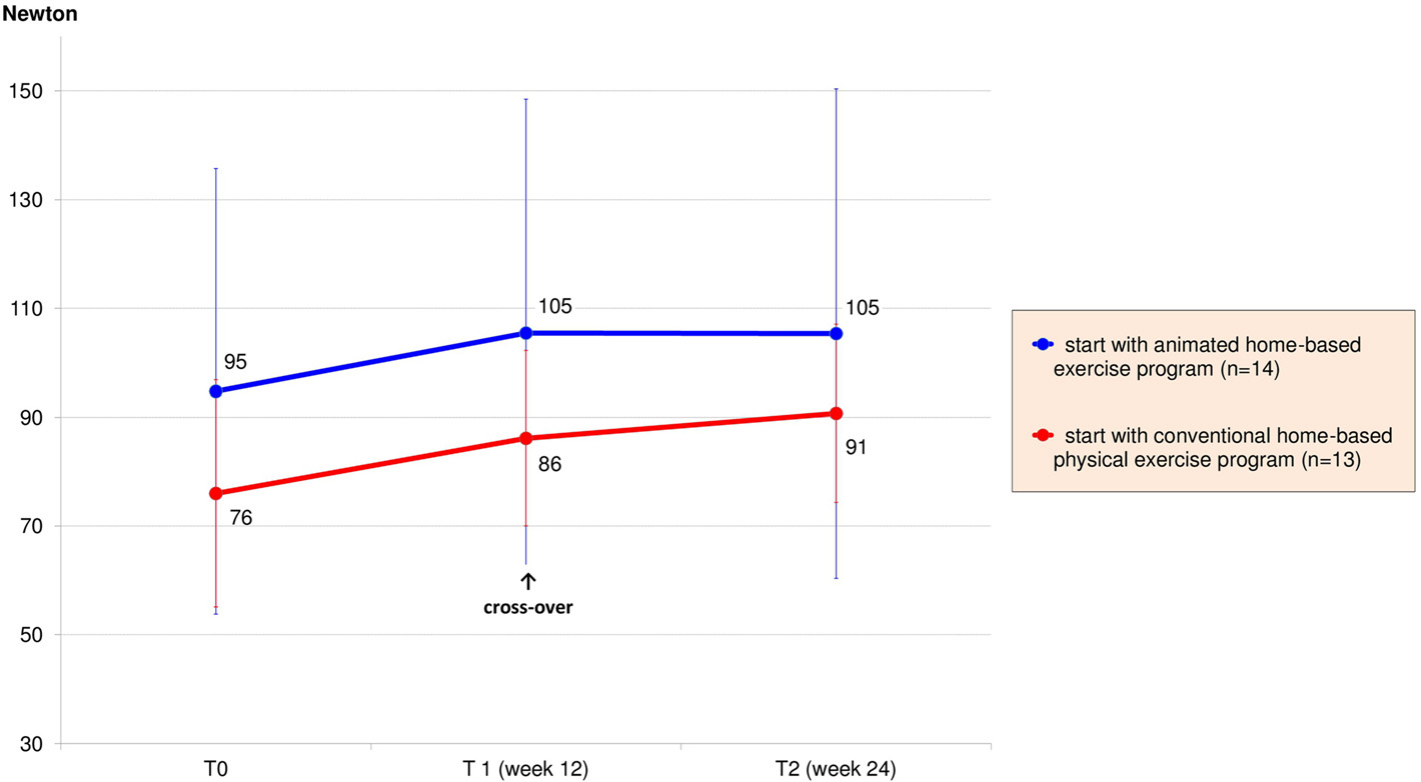
Results of muscle strength measurement

Assessments at week 12 and week 24 showed a benefit from both exercise programs. The analysis of tests showed no significant differences between both treatment arms after 12 weeks: Patient’s muscle strength was significantly improved by a mean value of 10 (SD ± 12) Newton (p < 0.001) representing an increase of +12 %, see also Fig. 4. The difference on improvement between both groups of 1.8 Newton was low and non-significant (*p* = 0.737). A detailed analysis of each muscle group is provided in Additional file 1.

**Fig. 5:**
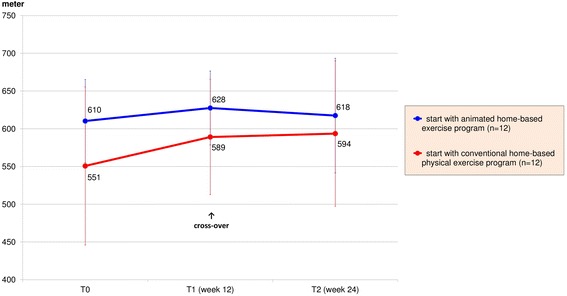
Results of 6-minute walk test

Of note, a clinically important improvement of the mean 6-MWT was observed. As shown in Fig. 5, the mean distance was increased by 28 (SD ± 64) meters after the first 12 weeks of treatment representing an increase of +5 % (*p* = 0.044). The difference of improvement between both groups of 9 m was without relevance and non-significant (*p* = 0.694). Results of respiratory tests showed no effects after 12 weeks. The peak expiratory flow at week 12 remained almost unchanged at 393 (SD ± 101, *p* = 0.965) liter/ minute on average with a difference of improvement of 12 liter/ minute between both groups (*p* = 0.569).

Week 24 results suggested that the sequence of applications was of minor relevance. The patients who started with the conventional home-based physical exercises and then crossed-over to the animated exercise program at week 12 had a mean improvement of muscle strength of 15 Newton (+20 %, *p* = 0.012) at the end of study. In comparison, the group with the other treatment order achieved an increase of 10 Newton (+11 %, *p* = 0.012). Analysis of Co-Variance of week 24-results and adjusted for baseline status showed a higher difference between both groups at week 24 (2.8 Newton, *p* = 0.670) than group-differences observed at week 12 (1.8 Newton, *p* = 0.737). Analysis of the 6-MWT disclosed similar results: at week 24 and compared to baseline, the study group that started with conventional physical exercises and subsequent animated game console program improved by a mean value of 7.8 % (*p* = 0.096) compared to 1.3 % (*p* = 0.767) of the other treatment sequence (9.1 m difference between groups; *p* = 0.782, Figs. 4 and 5).

### Patient Reported Outcomes (PRO’s)

The mean changes of HAQ-DI, SF36 and PtGA results were without statistical significance between groups. Low variations of changes of PRO’s between both groups indicates an equal performance of both treatment strategies (Additional file 2).

### Evaluation of the game console’s diary

Twenty-three virtual diaries have been analyzed regarding exercise time and frequency in order to check response on the one hand and feasibility on the other hand. We observed a total practice time/patient of less than 2 h (non-responder) as well as daily activities with more than 73 h exercising time during the 12-week period (high-responder).14 patients exercised more than 12 h each (e.g. 3 × 20 min per week/patient), 10 patients practiced more than 18 h each (e.g. 3 × 30 min per week/patient) and 8 patients had a total exercising time more than 27 h each (e.g. 3 × 45 min per week/patient). The very best attendee, an elderly patient, performed the animated fitness program 81 times and had an overall of 4404 min exercising time during the 12-weeks treatment period (6 h per week on average).

## Discussion

To our best knowledge, this is the first study using the Wii® console for treatment of patients with rheumatoid arthritis in a controlled setting.

Results of this study show that such an approach was beneficial to RA patients. Compared to standard physical home exercises, similar effects on muscle strength improvement and 6-MWT difference in both treatment groups were observed. Therefore such an animated program may be an alternative or additional option for RA patients depending on their individual preferences.

In this study, feasibility of a home based exercise program for RA patients by using a freely available Wii game console was confirmed. The recorded long exercising time and the high frequency using the game console by the high-responders can be interpreted as definite indicators for feasibility and acceptability. In particular, following the patients’ reported advantages of the game console, it could be a valuable opportunity for additional physiotherapy-like exercises for interested RA patients. Of course, the analysis of more than 22 h of interview material revealed also points of criticism as well as disadvantages of the tool (e.g. occasionally painful or time-consuming). These statements underline the need for further investigations how to best implement this approach in daily life.

In agreement with the overall result of a review about exercises in RA by Stenström and Minor [24], in this study no change in patient’s disease activity (PtGA, DAS28) and patient’s reported outcomes (HAQ, SF36) was also observed. However, other important functional benefits were achievable in addition to treatment effects of biologics.

Our study was limited due to the predefined alternating enrollment mode. In this context, all five male patients started with the animated home-based exercise program. This circumstance resulted in a higher baseline level of the function tests of that group which was however statistically adjusted. A matched pairs strategy (e.g. gender, age, stage of RA) could be an important option for further studies. Due to the characteristic of the game console, the exercises in both treatment groups were not totally identical. To reduce this particular bias, we used a pre-defined compilation of 31 exercises for conventional physical exercises inclusive comparable categories to the animated program (strength training, coordination, joint mobility, and relaxation). Lastly, these results do not refer to treatment changes and individual changes of disease activity during the course of the study.

Specific reasons for the obtained differences between both treatment sequences were not identified, but these results possibly point out an enhanced motivation for exercise by an animated enjoyable program.

By interpreting the results of this study, it is important to keep in mind the mean age of the participants (Ø 56 years) as well as the relatively long mean duration of their RA (Ø 13 years). In this context, an exercise time of up to 6 h per week by using the game console seems to be an striking and impressive result. The assessed acceptability of the animated exercise program by study population may also implicate a feasibility for younger and early RA patients. Therefore, with respect to the initially mentioned aim to offer RA patients a variety of physical activity options [23], the Wii console opens up new perspectives.

## Conclusion

Test results and interview analysis of this study lead to the assumption that an animated home-based exercise program by using a Wii® game console might be one of the enjoyable physical activities to maintain or improve RA patients’ fitness. This study showed that such a program was beneficial to and feasible for RA patients. Moreover the motivating aspect of the game console plays an important role independently of patients’ age and disease duration. Therefore, our developed treatment program could be also a good option for elderly RA patients in advanced stages of disease. Compared to standard physical home exercises, similar effects were observed indicating that such an animated program may be an alternative supportive option for RA patients integrating fun into the patients’ treatment concepts.

## Abbreviations

6-MWT, six-minute walk test; ACR, American College of Rheumatology; bDMARD, biological disease modifying anti-rheumatic drug; cDMARD, conventional disease modifying anti-rheumatic drug; DAS28, disease activity score (28 joints); HAQ-DI, health assessment questionnaire; MCID, minimal clinically important difference; PRO’s, patient reported outcomes; PtGA, patient’s global assessment; QDA, qualitative data analysis; RA, rheumatoid arthritis; SD, standard deviation; SF36, short form 36-item questionnaire.

## Additional files

Additional file 1: Results of muscle strength measurement (in detail). (XLSX 15 kb) Additional file 2: Patient reported outcomes (questionnaires). (DOCX 746 kb)
