# Supplementary material for: A prospective pilot study to evaluate an animated home-based physical exercise program as a treatment option for patients with rheumatoid arthritis
Source: BMC Musculoskelet Disord. 2016 Aug 18;17:351. doi: 10.1186/s12891-016-1208-3 (PMC4990861; doi:10.1186/s12891-016-1208-3)

Supplement 2: ***Patient Reported Outcomes (PRO’s)***

Similar results of HAQ-DI, SF36 and patients global assessment of disease activity (PtGA) have been determined in this study for both groups (figure 6). The HAQ-DI score of 0.85 (±0.53) at baseline decreased non-significantly by 0.05 points (0.80 ±0.55; p=0.202) during the first 12 weeks and returned to baseline level at week 24 (0.85 ±0.52). A mean change of the SF-36 score of 67 (± 15) at baseline by 1 point at week 12 (68 ±15, p=0.339) and 4 points at week 24 (63 ±19, p=0.120) is also non-significant and without clinically importance. As frequently observed under physiotherapy, a minor to moderate worsening of PtGA by +2mm (±11; p=0.354) in mean at week 12 and +11mm (±24; p=0.021) at week 24 were observed. At week 12, we noticed a low variation of changes of PRO’s between both groups and corresponding p-values varied from p=0.756 (HAQ-DI) through p=0.567 (SF36) to p=0.337 (PtGA), indicating an equal performance in both treatment strategies.


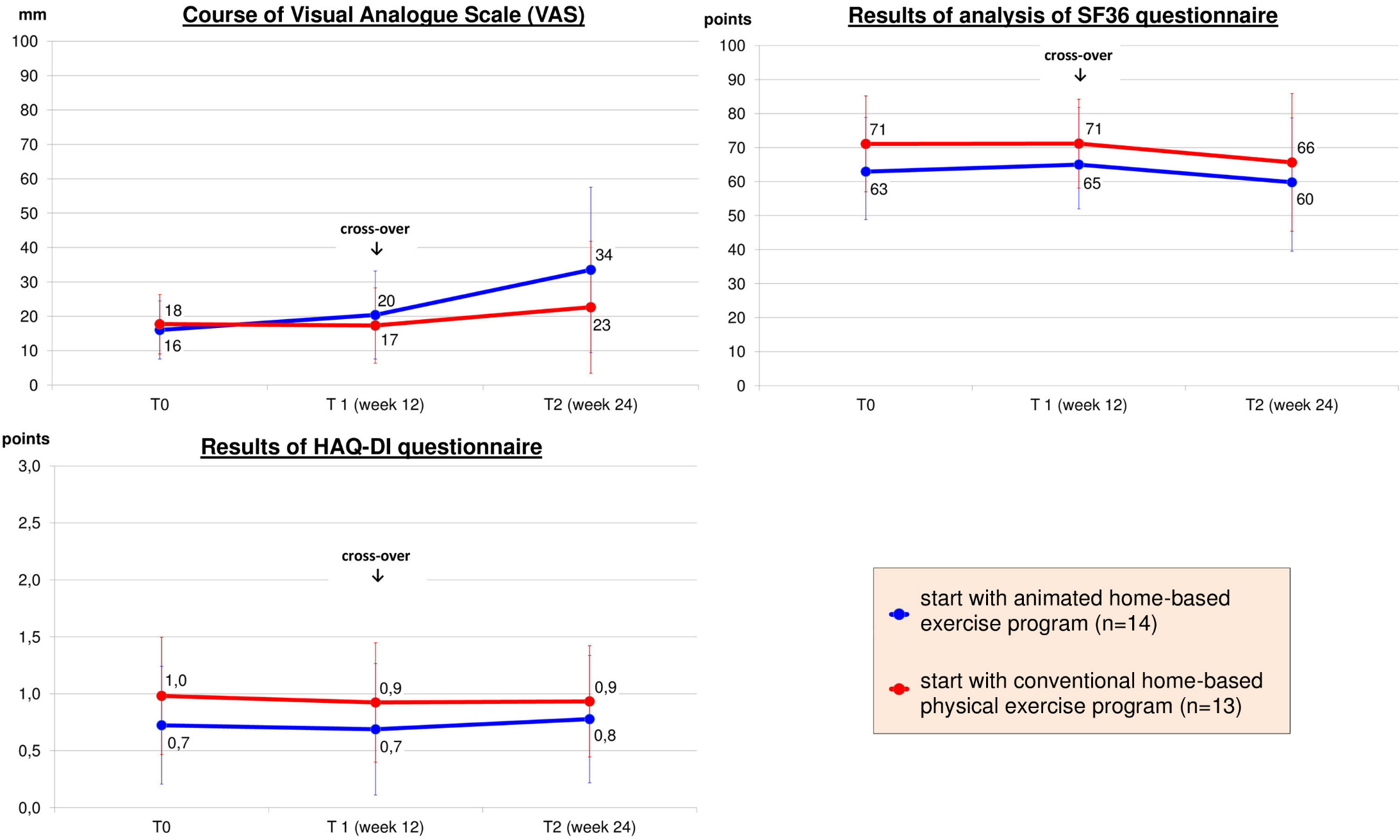

Supplement: Additional file 2: — Patient reported outcomes (questionnaires). (DOCX 746 kb) [file 12891_2016_1208_MOESM2_ESM.docx]
